# Targeting metabolic driving and intermediate influx in lysine catabolism for high-level glutarate production

**DOI:** 10.1038/s41467-019-11289-4

**Published:** 2019-07-26

**Authors:** Wenna Li, Lin Ma, Xiaolin Shen, Jia Wang, Qi Feng, Lexuan Liu, Guojun Zheng, Yajun Yan, Xinxiao Sun, Qipeng Yuan

**Affiliations:** 1 State Key Laboratory of Chemical Resource Engineering, Beijing University of Chemical Technology, Beijing, 100029 China; 2 School of Chemical, Materials and Biomedical Engineering, College of Engineering, The University of Georgia, Athens, GA 30602 USA

**Keywords:** Microbiology techniques, Applied microbiology, Metabolic engineering

## Abstract

Various biosynthetic pathways have been designed to explore sustainable production of glutarate, an attractive C5 building block of polyesters and polyamides. However, its efficient production has not been achieved in *Escherichia coli*. Here, we use *E. coli* native lysine catabolic machinery for glutarate biosynthesis. This endogenous genes-only design can generate strong metabolic driving force to maximize carbon flux toward glutarate biosynthesis by replenishing glutamate and NAD(P)H for lysine biosynthesis, releasing lysine feedback inhibition, and boosting oxaloacetate supply. We use native transporters to overcome extracellular accumulation of cadaverine and 5-aminovalerate. With these efforts, both high titer (54.5 g L^−1^) and high yield (0.54 mol mol^−1^ glucose) of glutarate production are achieved under fed-batch conditions. This work demonstrates the power of redirecting carbon flux and the role of transporters to decrease intermediate accumulation.

## Abstract (web summary)

The efficiency of producing glutarate, a valuable platform C5 compound, by engineered *E. coli* is low. Here, the authors achieve high titer and yield of glutarate production by pushing and pulling carbon flux in the native pathway and increasing intermediate influx using newly identified transporters.

## Introduction

Dicarboxylic acids are important building blocks for manufacturing polyesters and polyamides. Due to the concerns on oil supply and environment protection, increasing attention has been attracted to developing green and sustainable routes to these chemicals. Biosynthesis represents a promising alternative. Currently, bio-production of succinate is mature enough for commercial application and the titer reaches 152.2 g L^−1^ from glucose by recombinant *Corynebacterium glutamicum*^1^. Recently, production of adipic acid with high titer (68 g L^−1^) and yield (0.38 g g^−1^) has been achieved via a reversal β-oxidation pathway^2^. Glutarate is another important dicarboxylic acid used to manufacture polymers such as nylon-4, 5 and nylon-5, 5. The lowest melting point of glutarate among aliphatic dicarboxylic acids may endow the resultant polymers with unique properties. In addition, glutarate is a synthetic precursor of 1, 5-pentanediol, a common plasticizer and a precursor of polyesters^3^.

**Fig. 1:**
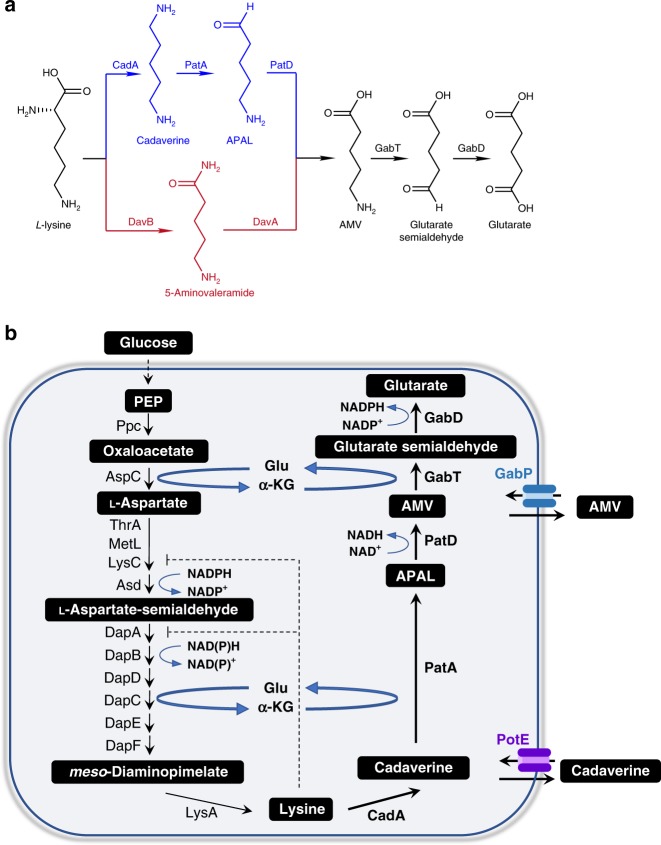
Glutarate biosynthesis via lysine catabolism. **a** The Cadaverine pathway (blue color) and the AMV pathway (red color) from lysine to glutarate. **b** *E. coli* native pathway for glutarate biosynthesis. Thick arrows indicate gene overexpression. Enzymes: CadA, lysine decarboxylase; DavA, delta-aminovaleramidase; DavB, lysine 2-monooxygenase; GabD, succinate-semialdehyde dehydrogenase; GabT, 4-aminobutyrate-2-oxoglutarate transaminase; PatA, putrescine aminotransferase; PatD, aminobutyraldehyde dehydrogenase. Metabolites: Glu, glutamate; α-KG, α-ketoglutarate; PEP, phosphoenolpyruvate; APAL, 5-aminopentanal; AMV, 5-aminovalerate

Over the years, several pathways have been designed for glutarate biosynthesis via 2-oxogularate reduction^4^, reverse β-oxidation from acetyl-CoA and malonyl-CoA^5^, carbon chain extension and decarboxylation from 2-oxogularate^6^, and lysine catabolism^4,7^. Among them, the lysine catabolism routes have the highest reported titers. Lysine can be degraded to glutarate via two partially different pathways, 5-aminovalerate (AMV) pathway and Cadaverine pathway^8,9^ (Fig. 1a). In AMV pathway, lysine is converted to AMV by the sequential catalysis of lysine 2-monooxygenase and delta-aminovaleramidase. AMV is further converted to glutarate by 5-aminovalerate transaminase and glutarate semialdehyde dehydrogenase. In Cadaverine pathway, conversion of lysine to AMV is catalyzed alternatively by lysine decarboxylase, cadaverine aminotransferase, and aminovaleraldehyde dehydrogenase. *C. glutamicum* has been extensively investigated for lysine production^10,11^. Recently, both AMV and Cadaverine pathways have been constructed in lysine-overproducing *C. glutamicum* strains^4,7,12^. The highest reported titer reached 90 g L^−1^ with the yield to be 0.28 mol mol^−1^ in the growth phase and 0.70 mol mol^−1^ in the production phase, respectively^12^. *E. coli* is another commonly used host for metabolic engineering. However, reconstructing AMV pathway in a lysine-overproducing *E. coli* strain resulted in only 0.82 g L^−1^ of glutarate in shake flasks, representing 9.1% of the theoretical yield (0.75 mol mol^−1^ glucose)^13^. The possible reasons are that (1) the pathways have unresolved limiting factors preventing carbon flux from reaching the end product and (2) the introduction of the downstream pathway perturbs the existing metabolic balance.

In fact, *E. coli* contains a native Cadaverine pathway, which is regulated by catabolite repression and induced by nitrogen limitation^14,15^. In this study we aim to engineer *E. coli* into an efficient glutarate producer by modulating its own genes. From oxaloacetate the full pathway involves 15 enzymatic steps (Fig. 1b), which represents a great challenge to achieve enhanced and balanced expression. Driving forces are essential for efficient biosynthesis of a target compound. They can be present as a push (e.g., increasing precursor supply), a pull (e.g., efflux or removal of the end product) or an internal recycle pump (e.g., redox balance). Under anaerobic conditions, NADH is frequently used as an internal recycle pump for the production of lactate, succinate, ethanol, and 1-butanol^16–19^. Under aerobic conditions, overexpressing key pathway enzymes and product exporters are usually used as the driving forces^20^. However, for long pathways, overexpression of multiple genes often causes metabolic burden and requires careful balance via strategies like multivariate modular optimization^21,22^. As indicated by previous studies, boosting lysine supply is not guaranteed for efficient glutarate production.

After analyzing the glutarate biosynthetic pathway, we find that at least three different driving forces (recycle of glutamate, recycle of redox power, and release of feedback inhibition) can be harnessed to continuously drag carbon flux through the target pathway. *E. coli* Cadaverine pathway involves one decarboxylation (CadA), two transamination (PatA and GabT), and two dehydrogenation reactions (PatD and GabD) (Fig. 1). Although it is one-step longer than AMV pathway, Cadaverine pathway requires no participation of molecular oxygen, which is advantageous for large-scale cultivation. Furthermore, per lysine consumed, the transamination reactions generate two glutamate and the dehydrogenases produce two NAD(P)H, respectively (Fig. 1). Interestingly, starting from oxaloacetate, the lysine biosynthetic pathway consumes two glutamate and two NAD(P)H to produce one lysine. Therefore, lysine catabolism could replenish glutamate and NAD(P)H for lysine biosynthesis. In addition, lysine consumption could naturally release its feedback inhibition on upstream genes and enzymes, representing as another driving force.

As expected, the synergistic use of these driving forces significantly improves production efficiency. In addition, extracellular accumulation of two pathway intermediates is observed and the problem is solved by identifying and expressing specific transporters. Further boosting oxaloacetate supply by activating glyoxylate cycle generates a final strain that produces glutarate with the highest reported titer and yield in shake flasks. This study provides a convenient and effective strategy for metabolic engineering of other long pathways.

## Results

### Pathway assembly using endogenous enzymes

*E. coli* native genes *cadA*, *patAD*, and *gabTD* were used to construct the glutarate biosynthetic pathway. Gene *cadA* encodes an inducible lysine decarboxylase. Compared with its constitutive counterpart LdcC, CadA shows higher activity and thermal stability^23^. PatA and PatD are annotated as putrescine aminotransferase and γ-aminobutyraldehyde dehydrogenase, and GabT and GabD as 4-aminobutyrate aminotransferase and succinate-semialdehyde dehydrogenase, respectively. These four enzymes show promiscuous activity and are involved in putrescine and cadaverine catabolism^4,24,25^.

**Fig. 2:**
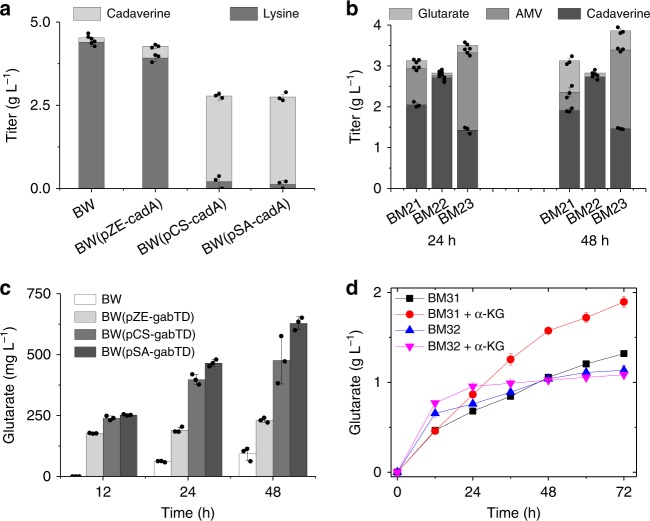
Modular optimization of glutarate production from lysine. Optimization of **a** cadA module, **b** patAD module, and **c** gabTD module. **d** Glutarate production from lysine. α-KG, α-ketoglutarate. Data shown are mean ± SD (*n* = 3 independent experiments). Source data are provided as a Source Data file

Based on the functional relevance, these genes were separated into three modules (cadA, patAD, and gabTD) and their expression was modulated stepwise using plasmids with different copy numbers. The results of feeding experiments showed that the low- and medium-copy plasmids (pSA-cadA and pCS-cadA) are more suitable for carrying cadA module than the high-copy plasmid (pZE-cadA). Strains BW (pSA-cadA) and BW (pCS-cadA) effectively converted lysine to cadaverine with the titers of 2.62 ± 0.07 (mean ± SD, same for the following) g L^−1^ and 2.57 ± 0.13 g L^−1^, respectively while the titer by strain BW (pZE-cadA) was only 0.35 ± 0.08 g L^−1^ (Fig. 2a). The control strain also produced tiny amount of cadaverine (0.13 ± 0.01 g L^−1^) due to the basal activity of the lysine decarboxylases (Fig. 2a).

Similarly, patAD module was optimized using cadaverine as the substrate. However, no obvious conversion occurred with all the three strains. To check the activity of PatA and PatD, 6× His-tagged proteins were purified and the result of in vitro assays showed that both enzymes were functionally expressed and the specific activities were 84.40 min^−1^ and 11.99 min^−1^, respectively. Accordingly, we speculated that the conversion is hindered by inefficient transport of cadaverine across the cell membrane. To verify this, patAD module was co-expressed with the optimized cadA module and lysine was used as the feeding substrate instead of cadaverine. The resultant strains successfully produced AMV. Strain BM23 carrying pSA-cadA and pCS-patAD produced the highest amount of AMV (1.93 ± 0.04 g L^−1^) at 48 h; notably, strain BM21 carrying pSA-patAD-cadA already accumulated as high as 0.77 ± 0.05 g L^−1^ of glutarate (Fig. 2b).

**Fig. 3:**
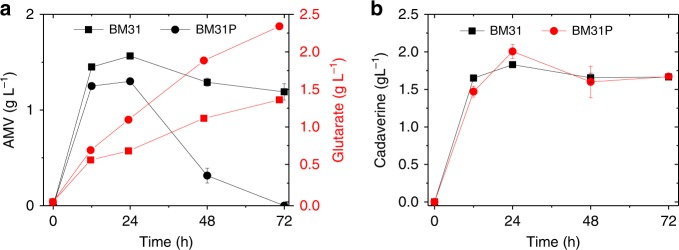
Overexpression of *gabP* decreases AMV accumulation. Effect of *gabP* expression on the accumulation of **a** AMV and glutarate, and **b** cadaverine. Data shown are mean ± SD (*n* = 3 independent experiments). Source data are provided as a Source Data file

When optimizing gabTD module, we observed a similar phenomenon as patAD module. When fed with 5 g L^−1^ of AMV, the strain carrying pSA-gabTD performed best but produced only 0.63 ± 0.03 g L^−1^ of glutarate, even lower than that produced by strain BM21 (0.77 g L^−1^) (Fig. 2c). Module gabTD was then grafted to the optimized cadA and patAD modules, resulting in two strains BM31(BW/pSA-cadA and pCS-patAD-gabTD) and BM32 (BW/pSA-cadA-gabTD and pCS-patAD). Slightly better than BM32, strain BM31 produced 1.32 ± 0.03 g L^−1^ of glutarate at 72 h in the feeding experiment. Furthermore, adding 2 g L^−1^ of α-ketoglutarate (α-KG) improved the titer to 1.90 ± 0.06 g L^−1^, suggesting that α-KG supplement is beneficial to the transamination reactions (Fig. 2d). In addition, we observed extracellular accumulation of both cadaverine (1.66 ± 0.04 g L^−1^ at 72 h) and AMV (1.19 ± 0.08 g L^−1^ at 72 h) (Fig. 3a, b), indicating that the re-uptake of these intermediates is rate-limiting. To solve this problem, specific transporters were screened and expressed in the next steps.

### GabP can efficiently import AMV

In *E. coli* chromosome, *gabP* is clustered with *gabT* and *gabD* into an operon. GabP is annotated as a 4-aminobutyrate transporter. Considering the promiscuous role of GabT and GabD in degradation of cadeverine and putrescine^4,24^, we speculate that GabP may also be able to transport AMV.

To explore the potential of GabP for AMV importation, strain BM31P (BW/pSA-cadA and pCS-patAD-gabTDP) was constructed and used for the feeding experiment. The result showed that extracellular amount of AMV first increased to 1.30 ± 0.01 g L^−1^ at 24 h and thereafter decreased significantly to non-detectable at 72 h, while the control strain BM31 accumulated 1.56 ± 0.04 g L^−1^ of AMV at 24 h and the amount decreased slightly to 1.19 ± 0.08 g L^−1^ at 72 h (Fig. 3a). The result indicates that GabP indeed is a functional AMV transporter and increasing its expression can reduce extracellular accumulation of AMV. Consequently, glutarate titer was significantly improved from 1.36 ± 0.03 g L^−1^ to 2.34 ± 0.01 g L^−1^ (Fig. 3a). Recently, it was shown that expression of an importer is also crucial for efficient re-uptake of 5-aminovalerate in *C. glutamicum*.^12^ However, the problem of cadaverine accumulation remained unsolved (Fig. 3b).

### PotE is a bi-directional cadaverine transporter

Theoretically, extracellular accumulation of cadaverine can be decreased by either preventing its efflux or enhancing its uptake. However, it seems challenging to implement the first strategy, because cadaverine can be exported via multiple potential exporters/antiporters including CadB, SapB, PotE, and yet un-characterized ones. CadB is both cadaverine importer and a lysine:cadaverine antiporter^26^, SapB is a subunit of the newly characterized putrescine exporter SapBCDF^27^, and PotE is a bi-directional putrescine transporter^28^. To validate their effect on cadaverine export, three single knockout strains (BWΔ*cadB*, BWΔ*sapB*, and BWΔ*potE*) were transformed with plasmids pSA-cadA and pCS-patAD-gabTDP, and the resultant strains were fed with 5 g L^−1^ of lysine. The results showed that the single knockouts had no positive effect on glutarate production and knocking out *potE* or *sapB* even caused impaired cell growth and decreased glutarate production (Supplementary Fig. 1). Thus, the performance of multiple knockouts was not further tested and the second strategy was adopted instead.

**Fig. 4:**
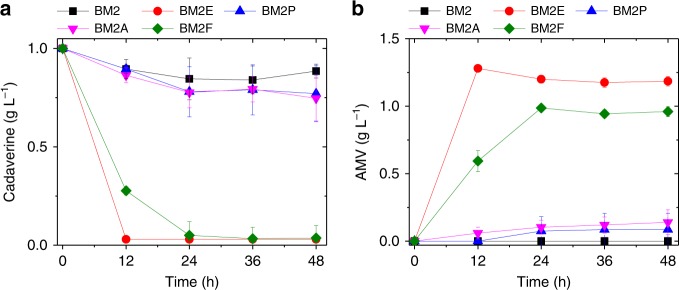
Comparison of the efficiency of four candidate cadaverine transporters. **a** Cadaverine consumption with time; **b** AMV accumulation with time. Data shown are mean ± SD (*n* = 3 independent experiments). Source data are provided as a Source Data file

*E. coli* contains multiple importers including PotE, PuuP, PotABCD, and PotFGHI^28–31^. However, their capabilities of transporting cadaverine have not been investigated before. Their encoding genes were cloned into plasmid pSA74. Co-transferring the resultant plasmids with pCS-patAD into strain BW25113 (BW, for short) generated four corresponding strains (BW2E, BW2P, BW2A, and BW2F), which were used for the feeding experiment. Among the four importers, PotE was shown to be the most effective and strain BM2E containing pSA-potE and pCS-patAD completely transported and converted 1 g L^−1^ of cadaverine into AMV within 12 h (Fig. 4a, b).

In order to further verify the function of PotE, strain BM31PE was constructed and cultured in M9 medium supplemented with 5 g L^−1^ of lysine. Compared with that of strain BM31P (1.60 ± 0.07 g L^−1^), strain BM31PE accumulated higher amount of extracellular cadaverine (2.04 ± 0.04 g L^−1^) at 12 h and then the amount decreased to 1.53 ± 0.01 g L^−1^ at 72 h, which is comparable to that of BM31P (1.56 ± 0.11 g L^−1^) (Supplementary Fig. 2). This result confirmed that PotE is a bi-directional cadaverine transporter. Under this condition, cadaverine is prone to be exported to decrease its intracellular accumulation.

### De novo biosynthesis of glutarate

**Fig. 5:**
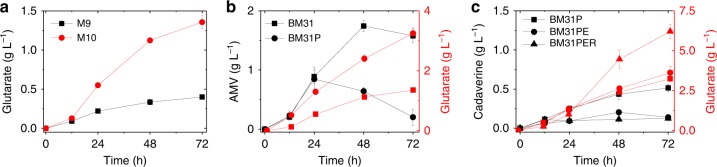
De novo biosynthesis of glutarate. **a** Comparison of glutarate production in M9 and M10 media. **b** Comparison of glutarate production and AMV accumulation by strains BM31 and BM31P. **c** Comparison of glutarate production and cadaverine accumulation by strains BM31P, BM31PE, and BM31PER. Data shown are mean ± SD (*n* = 3 independent experiments). Source data are provided as a Source Data file

To explore de novo biosynthesis of glutarate, strains BW and BM31 were cultivated in M9 medium with 10 g L^−1^ of glucose. *E. coli* BW produced non-detectable lysine and glutarate, while strain BM31 produced 0.40 g L^−1^ of glutarate at 72 h (Fig. 5a). This demonstrates that the enhancement of lysine catabolism generates expected driving forces. Glutarate biosynthesis involves multiple transamination reactions and the ammonia-rich M10 medium was used previously for glutarate production in *E. coli*^13^. Culturing strain BM31 in M10 medium increased glutarate titer to 1.36 ± 0.08 g L^−1^ (Fig. 5a). Meanwhile, 1.58 ± 0.12 g L^−1^ of AMV and 0.32 ± 0.03 g L^−1^ of cadaverine were accumulated (Supplementary Fig. 3). By contrast, strain BM31P produced 3.25 ± 0.18 g L^−1^ of glutarate, while extracellular AMV decreased to 0.20 ± 0.14 g L^−1^ (Fig. 5b), confirming that AMV influx is crucial for efficient glutarate production. Unlike in the feeding experiment, in de novo production overexpression of PotE showed positive effect on cadaverine importation, leading to a slight increase in glutarate titer (3.62 ± 0.28 g L^−1^) by strain BM31PE (Fig. 5c).

### Boosting oxaloacetate supply enhances glutarate production

With the above efforts, glutarate production was improved stepwise. However, glutarate yield (0.20 mol mol^−1^) by strain BM31PE is still far below the theoretical value. We then attempted to enhance glutarate production by overexpressing key upstream genes including phosphoenolpyruvate carboxylase, aspartate kinase, and 4-hydroxy-tetrahydrodipicolinate synthase, which has been shown to be effective in increasing production of lysine and its derivatives^32–34^. However, the resultant strains produced less amount of glutarate than strain BM31PE (Supplementary Fig. 4), suggesting that the prior balance may be disturbed. Instead of modulating expression of these genes, we turned to enhance oxaloacetate supply by deleting *iclR*. IclR is a transcription factor that represses expression of the glyoxylate bypass operon^34^. Glyoxylate bypass is a common mechanism of replenishing oxaloacetate consumed for biosynthesis. Strikingly, deletion of *iclR* led to a significant increase in glutarate production. The titer by strain BM31PER reached 6.21 ± 0.44 g L^−1^ (0.34 mol mol^−1^) at 72 h. The result demonstrates that boosting oxaloacetate supply provided another strong driving force.

### Confirming the importance of driving forces

To further demonstrate the importance of driving forces, we constructed three strains BWL0 (BWΔ*cadA*Δ*ldcC*), BWL1 (BWΔ*patA*/pSA-cadA), and BWL3 (BWΔ*gabT*/pSA-cadA and pCS-patAD) to produce lysine, cadaverine, and AMV, respectively. As expected, the titers of the corresponding products increased with the extension of the pathway (Table 1). Under normal conditions, lysine biosynthesis is tightly regulated. Strain BWL0 produced non-detectable lysine because of the lack of driving forces. Strain BWL1 produced 1.52 ± 0.05 g L^−1^ of cadaverine, indicating that converting lysine to cadaverine creates driving force by releasing feedback inhibition on upstream genes and enzymes. Furthermore, strain BWL3 produced 3.44 ± 0.15 g L^−1^ of AMV, confirming that the transamination and dehydrogenation steps provided additional driving forces. Further extending the pathway resulted in a minor increase glutarate titer (3.62 ± 0.28 g L^−1^) by BM31PE, suggesting that there may be a compromise between the pathway length and the driving force strength. In addition, two control strains (BWL5/C1 and C2) were also constructed. As noticed above, knocking out *potE* caused unhealthy cell growth. Therefore, strain BWL5/C1 with *potE* and *gabP* double knockouts showed both decreased glutarate production and increased AMV accumulation. Strain BWL5/C2 with the basal expression of CadA produced only 0.91 ± 0.12 g L^−1^ of glutarate. These results indicate that the existence of limiting steps could weaken the effectiveness of the driving forces.

**Table 1:** Verification of the effect of driving forces on the pathway efficiency

Strains	Description	Lysine (g L^−1^)	Cadaverine (g L^−1^)	AMV (g L^−1^)	Glutarate (g L^−1^)
BWL0	BWΔ*cadA*Δ*ldcC*	ND*	ND	ND	ND
BWL1	BWΔ*patA*/pSA-cadA	ND	1.52 ± 0.05**	0.17 ± 0.02	ND
BWL3	BWΔ*gabT*/pSA-cadA, pCS-patAD	ND	0.67 ± 0.12	3.44 ± 0.15	ND
BWL5 (BM31PE)	BW/pSA-cadA-potE, pCS-patAD-gabTDP	ND	0.12 ± 0.07	0.03 ± 0.01	3.62 ± 0.28
BM31PER	BWΔ*iclR*/pSA-cadA-potE, pCS-patAD-gabTDP	ND	0.12 ± 0.02	0.32 ± 0.11	6.21 ± 0.44
BWL5/C1	BWΔ*gabP*Δ*potE*/pSA-cadA, pCS-patAD-gabTD	ND	ND	1.44 ± 0.08	0.73 ± 0.12
BWL5/C2	BW/pCS-patAD-gabTDP	ND	ND	ND	0.91 ± 0.12

^*^ND not detected

^**^Data shown are mean ± SD (*n* = 3 independent experiments)

### Fed-batch production of glutarate

**Fig. 6:**
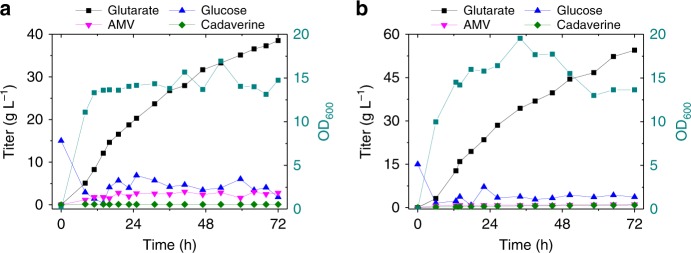
Fed-batch production of glutarate in 3 L bioreactors. Strains BM31PE (**a**) and BM31PER (**b**) were used for the experiments, respectively. The minimum threshold of DO was set at 10%. The result of a duplicate experiment is shown in Supplementary Fig. 6, which results in similar titer and yield. Source data are provided as a Source Data file

To evaluate the scale-up potential of glutarate production, fed-batch experiments were carried out in bioreactors using strains BM31PE and BM31PER. We investigated the effect of dissolved oxygen (DO) on glutarate production. The results showed that high DO is benefit for cell growth but not for glutarate accumulation. When DO was set at 20%, the cell density (OD_600_) of strain BM31PE reached 50, while the final titer of glutarate was only 8 g L^−1^ from 137 g L^−1^ glucose (Supplementary Fig. 5). On the contrary, at 10% set DO, the cells entered the stationary phase within 12 h and the OD_600_ value fluctuated around 15 till the end of the cultivation (Fig. 6a). Interestingly, glutarate was produced continuously independent of cell growth, demonstrating the advantage of interior balance of the pathway. At 72 h, 38.5 g L^−1^ (0.48 mol mol^−1^) of glutarate was produced with the consumption of 111 g L^−1^ of glucose. Both AMV and cadaverine were accumulated at low levels. Acetate was the main byproduct, which accumulated 16 g L^−1^ by the end of cultivation. When strain BM31PER was used, it produced 54.5 g L^−1^ of glutarate at 72 h and the yield (0.54 mol mol^−1^) reached 72% of the theoretical maximum (Fig. 6b).

## Discussion

In order to adapt to the changing environment, microorganisms contain various degradation pathways, in which may exist some useful intermediates, e.g., muconic acid^35,36^ and maleic acid^37^ in aromatic compound degradation. Connecting a degradation pathway with the corresponding biosynthetic pathway can lead to de novo biosynthesis of the target compound. In *E. coli*, lysine decarboxylation to cadaverine is involved in its adaption to acid stress^38^. Cadaverine can be re-assimilated into the TCA cycle via further enzymatic steps^9^. In this study, we used *E. coli* native lysine biosynthetic and catabolic mechanism for glutarate production without introducing foreign genes.

The long distance from the central metabolism means that only limited flux could be allocated to glutarate under normal conditions. In previous studies, lysine-overproducing strains are commonly used to ensure enough precursor supply. For example, lysine-hyperproducing strain *C. glutamicum* LYS-12 was used as the chassis strain. With 18 genomic changes the best strain produced 1.85 g L^−1^ (0.27 mol mol^−1^) of glutarate in shake flasks and 90 g L^−1^ of glutarate under fed-batch conditions. In this study, the multiple driving forces were synergistically used, instead of enhancing lysine biosynthesis, to pump the metabolic flux for glutarate production. This strategy only requires optimized expression of the Cadaverine pathway and the corresponding transporter genes, together with the deletion of one regulator gene. The final strain produced 6.21 g L^−1^ (0.34 mol mol^−1^) of glutarate in shake flasks. However, under fed-batch conditions, the titer reached 54.5 g L^−1^, lower than that obtained by *C. glutamicum* (90 g L^−1^). It is reported that *C. glutamicum* can grow in the presence of up to 60 g L^−1^ of glutarate^12^, whereas the growth of *E. coli* is severely inhibited at 20 g L^−1^ of glutarate^13^. Therefore, the strain tolerance is a major limitation for glutarate production in *E. coli*, which could be solved by adaptive laboratory evolution.

Cell membrane is selective permeable and transportation of many substances are facilitated by specific transporters. Transporter engineering has been successfully used for co-utilization of mixed carbon sources^39^ and enhancement of product efflux^20^. However, there are few reports on expressing transporters to reduce extracellular accumulation of pathway intermediates. In an example, a co-culture system was designed to produce muconic acid from a glucose/xylose mixture and the permease ShiA was expressed in the downstream strain to achieve efficient transport and assimilation of the intermediate 3-dehydroshikimic acid.^40^ Cadaverine and AMV are two intermediates in glutarate biosynthesis. In this study, extracellular accumulation of these two compounds was diminished by screening and expressing specific transporters, which is an essential step toward efficient glutarate production.

## Methods

### Experimental materials

Strains and plasmids used in this study are listed in Supplementary Table 1 and Supplementary Table 2, respectively. Primers designed were listed in Supplementary Table 3. *E. coli* XL-1 Blue was used as the host for plasmid construction. *E. coli* BW and its derived strains were used for feeding experiment and de novo production. *E. coli* strain BL21 Star (DE3) was used for protein expression and purification. Plasmids pZE12-luc (high-copy number), pCS27 (medium-copy number), and pSA74 (low-copy number) were used for pathway construction^41^. Plasmid pETDuet-1 was used as the vector for expression and purification of PatA and PatD. Plasmids were constructed by standard enzyme digestion and ligation. Point mutations were introduced by overlap PCR. BWΔ*cadB*, Δ*sapB*, and Δ*potE* knockout strains were purchased from Coli Genetic Stock Center. The other knockout strains were constructed by RED recombination following the standard protocols^42^.

### Culture media and conditions

Luria–Bertani (LB) medium containing yeast extract (5 g L^−1^), tryptone (10 g L^−1^), and NaCl (10 g L^−1^) was used for inoculant preparation and cell propagation. M9 medium containing glucose (10 g L^−1^), yeast extract (2 g L^−1^), NH_4_Cl (1 g L^−1^), Na_2_HPO_4_ (6.78 g L^−1^), KH_2_PO_4_ (3 g L^−1^), MOPS (morpholinepropanesulfonic acid, 2 g L^−1^), NaCl (0.5 g L^−1^), MgSO_4_ (1 mM), and CaCl_2_ (0.1 mM) was used for feeding experiments. M10 medium containing glucose (25 g L^−1^), yeast extract (2 g L^−1^), (NH_4_)_2_SO_4_ (16 g L^−1^), KH_2_PO_4_ (1 g L^−1^), MgSO_4_ (1 g L^−1^), CaCO_3_ (30 g L^−1^), and CaCl_2_ (0.1 mM) was used for de novo production.

For shake flask experiments, single fresh colonies were inoculated into 4 ml of LB media and grown overnight at 37 °C. Subsequently, overnight cultures (1 ml) were transferred to 50 mL of fresh M9 or M10 media, grown at 37 °C and 200 r.p.m. for 2 h and then induced with 0.5 mM isopropyl-β-d-thiogalactoside (IPTG). The induced cultures continued to grow at 30 °C and 200 r.p.m. Ampicillin, kanamycin, and chloramphenicol were added to the medium when necessary at final concentrations of 100, 50, and 34 μg mL^−1^, respectively. Samples were taken at regular time intervals for analysis of cell growth and product accumulation.

For the fed-batch cultivation, strain BM31PE and BM31PER was cultivated in 3 L bioreactors containing 1 L of modified M10 medium. The initial medium (per liter) contains the following: glucose (15 g), (NH_4_)_2_SO_4_ (8 g), KH_2_PO_4_ (6 g), Na_2_HPO_4_ (1 g), yeast extract (10 g), MgSO_4_ (2 g), citric acid (0.8 g), V_B1_ (30 mg), V_B6_ (30 mg), and 5 mL of trace element solution (10 g FeSO_4_·7H_2_O, 2 g CaCl_2_, 2.2 g ZnSO_4_·7H_2_O, 0.5 g MnSO_4_·4H_2_O, 1 g CuSO_4_·5H_2_O, 0.1 g (NH_4_)_6_Mo_7_O_24_·7H_2_O, 0.23 g Na_2_B_4_O_7_·10H_2_O per liter). The seed culture was inoculated into the bioreactor at 8% and cultivated at 37 °C. When OD_600_ reached around 15, 0.5 mM IPTG was added to induce protein expression and then the temperature was shifted to 30 °C. Glucose concentration was maintained below 5 g L^−1^. The feeding solution (per liter) contains glucose (625 g), KH_2_PO_4_ (22.5 g), Na_2_HPO_4_ (3.75 g), and MgSO_4_ (2.5 g). The pH was maintained at 7.0 during the whole process. The cell growth was monitored by measuring the optical density at 600 nm (OD_600_) and the supernatants was subjected to high-performance liquid chromatography (HPLC) analysis.

### Assays of PatA and PatD

Plasmids pET-patA and pET-patD were transformed into *E. coli* BL21 Star (DE3), separately. The recombinant strains were cultured to an OD_600_ of 0.6 and induced with 0.5 mM IPTG for 12 h at 25 °C. Cells were collected and re-suspended in lysis buffer (50 mM Tris-HCl, 300 mM sodium chloride, 10 mM imidazole, pH 8.0). The His-tagged proteins were purified using Ni^+^-affinity chromatography and protein concentration was determined using the bicinchoninic acid (BCA) method.

For PatA assay, the reaction system contains 20 mM cadaverine, 20 mM α-ketoglutarate, and 0.075 mg of purified PatA in 100 mM Tris-HCl buffer (pH 8.5) with the final volume 1 mL. The reaction was maintained at 30 °C for 30 min. A coupled assay was used to estimate the activity of PatD. The system contains 4 mM 5-aminopentanal (produced from cadaverine by PatA), 4 mM NAD^+^, and 0.122 mg of purified PatD. Reactions were started by the addition of purified enzyme and maintained at 30 °C for 30 min. The activity of PatA and PatD were determined by measuring the consumption of cadaverine or the production of AMV, respectively.

### HPLC analysis of product and intermediates

Lysine, cadaverine, and AMV were analyzed by HPLC (HITACHI) equipped with a reverse-phase Diamonsil C18 column (Diamonsil 5 μm, 250 × 4.6 mm) and UV–VIS detector. Samples were centrifuged at 7700 × *g* for 10 min. The supernatant was reacted with phenyl isothiocyanate, filtered through 0.22 μm film, and used for HPLC analysis. Solvent A was methanol and solvent B was water with 0.1% formic acid. The column temperature was set at 40 °C. The following gradient was used at a flow rate of 1 mL min^−1^: 32% to 80% solvent A for 20 min, 80% to 32% solvent A for 2 min, and 32% solvent A for an additional 5 min. Quantification was based on the peak areas at specific wavelengths (254 nm). The analysis of glutarate and glucose was performed by HPLC (HITACHI) equipped with an Organic Acid Analysis Column (Amine HPX-87H Ion Exclusion Column, 300 mm × 7.8 mm) and refractive index detector. The mobile phase was 5 mM H_2_SO_4_ at a flow rate of 0.5 mL min^−1^ and the oven temperature was set at 60 °C. The product glutarate was analyzed by ESI-MS and the molecular weight was in accordance with that of the glutarate standard (Supplementary Fig. 7).

## Supplementary information

Supplementary Information

Reporting summary

Source Data

## Data Availability

Data supporting the findings of this work are available within the paper and its Supplementary Information files. A reporting summary for this Article is available as a Supplementary Information file. The datasets generated and analyzed during the current study are available from the corresponding author upon request. The source data underlying Figs. 2–6 and Supplementary Figs. 1–6 are provided as a Source Data file.
