# Supplementary material for: Targeting metabolic driving and intermediate influx in lysine catabolism for high-level glutarate production
Source: Nat Commun. 2019 Jul 26;10:3337. doi: 10.1038/s41467-019-11289-4 (PMC6659618; doi:10.1038/s41467-019-11289-4)
Supplement: Supplementary file 1 — Supplementary Information [file 41467_2019_11289_MOESM1_ESM.pdf]

Targeting metabolic driving and intermediate influx in lysine catabolism for high-level glutarate production

Li *et al.*

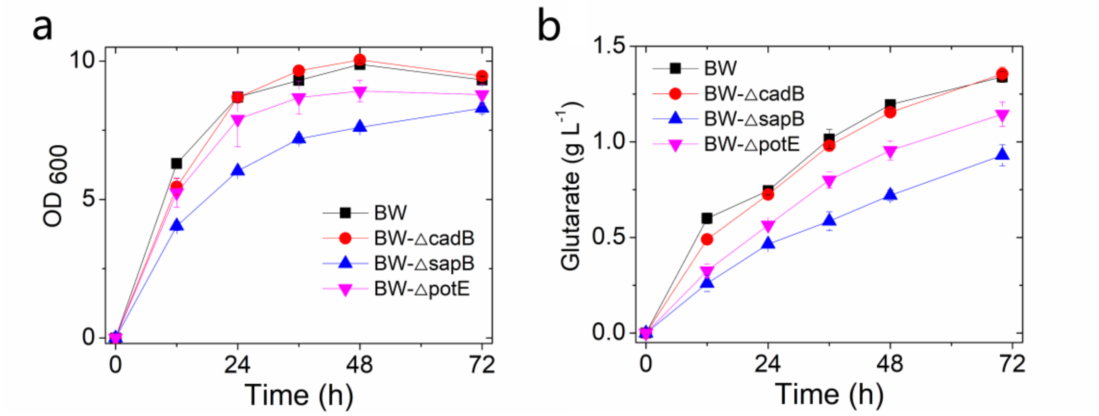

Supplementary Figure 1. Effect of knocking out of cadaverine efflux genes. (a) cell growth and **(b)** glutarate production. Data shown are mean ± s.d. (n = 3 independent experiments). Source data are provided as a Source Data file.

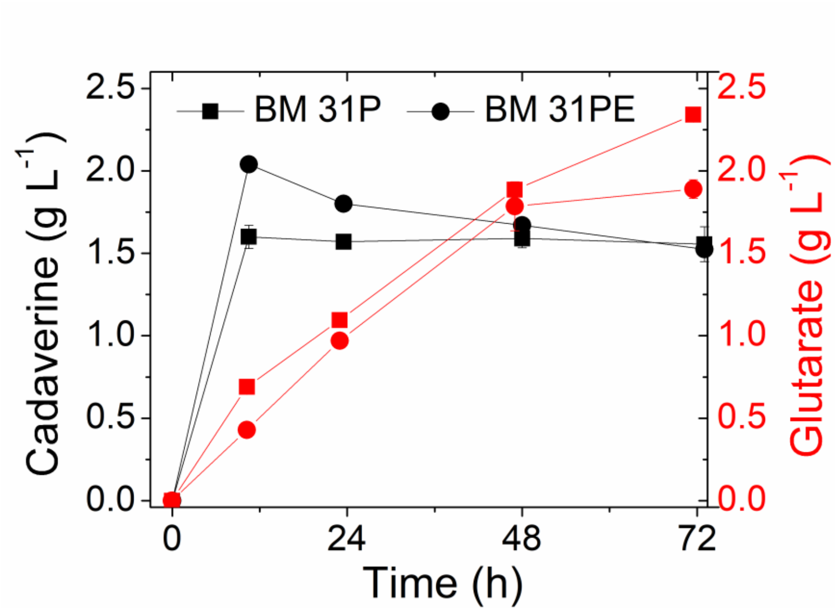

Supplementary Figure 2. Effect of expressing PotE on glutarate and cadaverine accumulation in the feeding experiment. Black curves indicate cadaverine; Red curves indicate glutarate. Data shown are mean ± s.d. (n = 3 independent experiments). Source data are provided as a Source Data file.

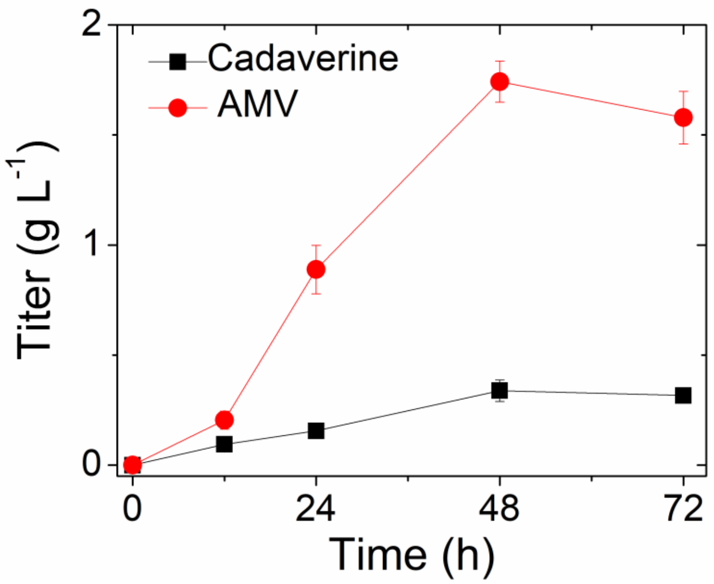

Supplementary Figure 3. Accumulation of cadaverine and AMV by BM31 strain cultured in M10 medium. Data shown are mean ± s.d. (n = 3 independent experiments). Source data are provided as a Source Data file.

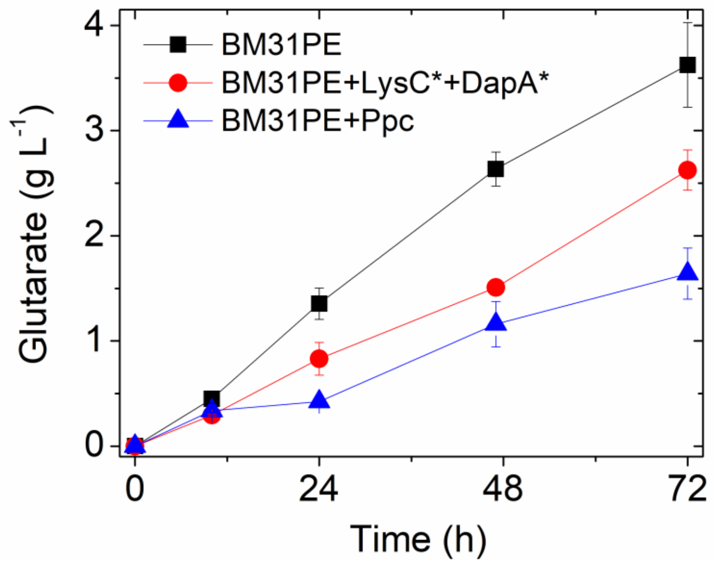

Supplementary Figure 4. Effect of key genes involved in lysine biosynthesis on glutarate production. Data shown are mean ± s.d. (n = 3 independent experiments). Source data are provided as a Source Data file.

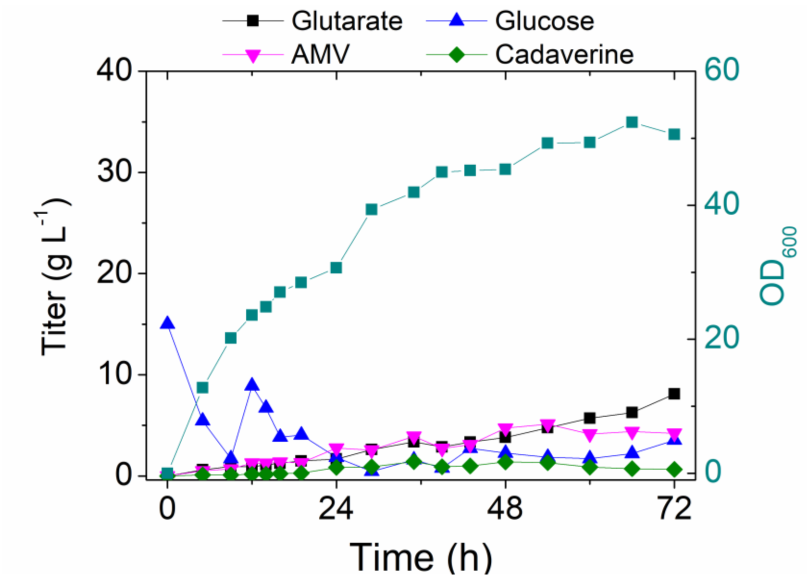

Supplementary Figure 5. Fed batch production of glutarate in a 3 L bioreactor. The minimum threshold of DO was set at 20 %. Source data are provided as a Source Data file.

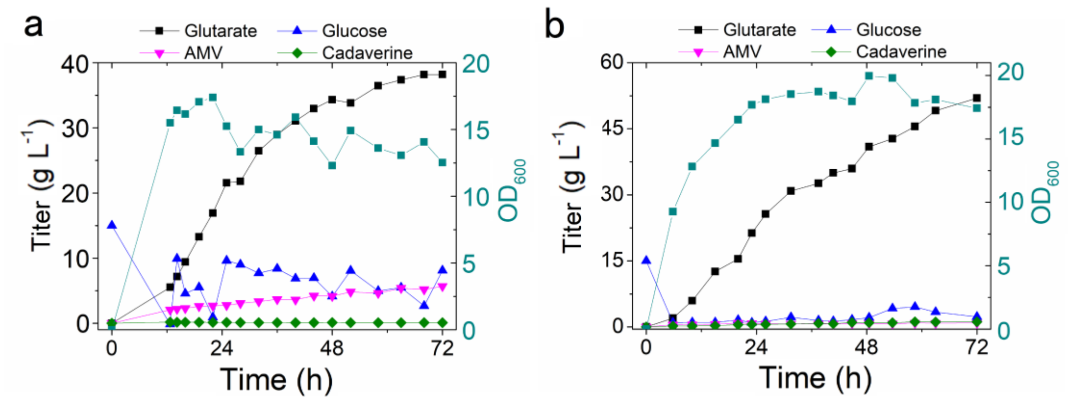

Supplementary Figure 6. Fed-batch production of glutarate in 3 L bioreactors. Strains BM31PE (a) and BM31PER (b) were used for the experiments, respectively (Batch 2). The minimum threshold of DO was set at 10 %. Source data are provided as a Source Data file.

a

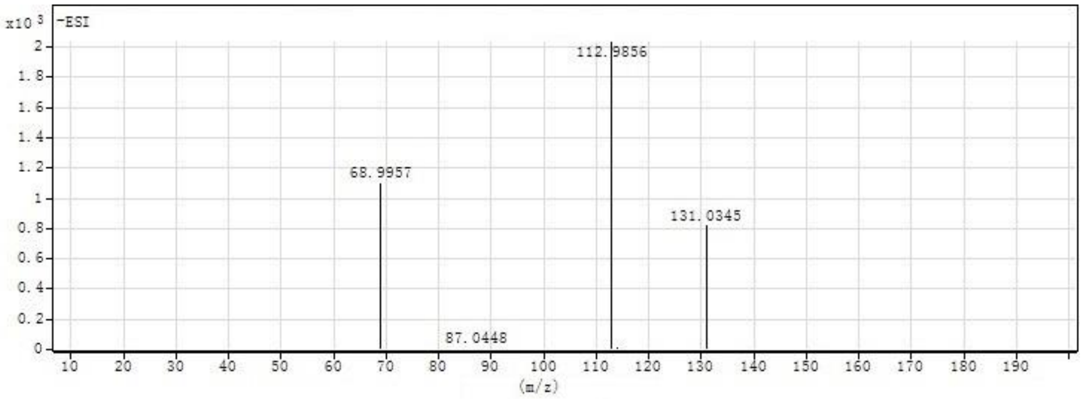

b

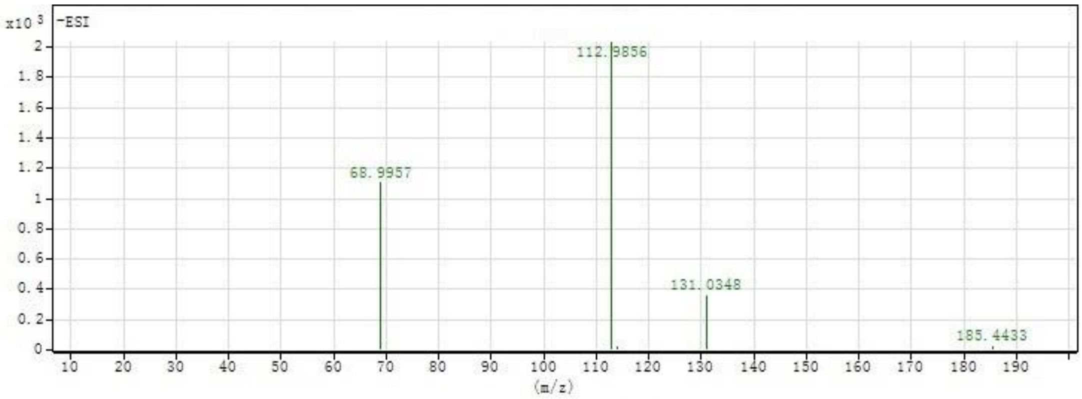

Supplementary Figure 7. ESI-MS results of glutarate. a Glutarate standard. **b** Glutarate sample.

Supplementary Table 1. Strains used in this study

**Strains**	**Description**	**Source**
E. coli BW25113	*rrnBT*14 Δ*lacZWJ*16 *hsdR*514 Δ*araBADAH*33 Δ*rhaBADLD*78	Coli genome stock center
E. coli XL-1 Blue	*recA1 endA1gyrA96thi-1hsdR17supE44relA1lac*	Stratagene
E. coli BL21 Star (DE3)	F^- *ompT* $hsdS_B$ $(r_B^- m_B^-)$ *gal dcm (DE3)*	Invitrogen
BW (pZE-cadA)	BW25113, pZE-cadA	This study
BW (pCS-cadA)	BW25113, pCS-cadA	This study
BW (pSA-cadA)	BW25113, pSA-cadA	This study
BM21	BW25113, pSA-patAD-cadA	This study
BM22	BW25113, pSA-cadA and pZE-patAD	This study
BM23	BW25113, pSA-cadA and pCS-patAD	This study
BW (pZE-gabTD)	BW25113, pZE-gabTD	This study
BW (pCS-gabTD)	BW25113, pCS-gabTD	This study
BW (pSA-gabTD)	BW25113, pSA-gabTD	This study
BM31	BW25113, pSA-cadA and pCS-patAD-gabTD	This study
BM32	BW25113, pSA-cadA-gabTD and pCS-patAD	This study
BM31P	BW25113, pSA-cadA and pCS-patAD-gabTDP	This study
BWΔ*cadB*	BW25113Δ*cadB*, pSA-cadA and pCS-patAD-gabTDP	This study
BWΔ*sapB*	BW25113Δ*sapB*, pSA-cadA and pCS-patAD-gabTDP	This study
BWΔ*potE*	BW25113Δ*potE*, pSA-cadA and pCS-patAD-gabTDP	This study
BM2	BW25113, pCS-patAD	This study
BM2E	BW25113, pCS-patAD and pSA-potE	This study
BM2P	BW25113, pCS-patAD and pSA-puuP	This study
BM2A	BW25113, pCS-patAD and pSA-potABCD	This study
BM2F	BW25113, pCS-patAD and pSA-potFGHI	This study
BM31PE	BW25113, pSA-cadA-potE and pCS-patAD-gabTDP	This study
BWL0	BW25113Δ*cadA*Δ*ldcC*	This study
BWL1	BW25113Δ*patA*, pSA-cadA	This study
BWL3	BW25113Δ*gabT*, pSA-cadA and pCS-patAD	This study
BWL5/C1	BW25113Δ*gabP*Δ*potE*, pSA-cadA and pCS-patAD-gabTD	This study
BWL5/C2	BW25113, pCS-patAD-gabTDP	This study
BM31PER	BW25113Δ*iclR*, pSA-cadA-potE and pCS-patAD-gabTDP	This study

Supplementary Table 2. Plasmids used in this study

**Plasmids**	**Description***	**Source**
pZE12-luc	$P_L$lacO1, *colE ori*, *luc*, *Amp*^{R}	Ref. 14
pCS27	$P_L$lacO1, *p15A ori*, *Kan*^{R}	Ref. 14
pSA74	$P_L$lacO1, *pSC101 ori*, *Cl*^{R}	Ref. 14
pETDuet-1	PT7, *pBR322 ori*, *Amp*^{R}	Novagen
pZE-cadA	pZE12-luc, *cadA*	This study
pCS-cadA	pCS27, c*adA*	This study
pSA-cadA	pSA74, *cadA*	This study
pZE-patAD	pZE12-luc, *patA* and *patD*	This study
pCS-patAD	pCS27, *patA* and *patD*	This study
pSA-patAD	pSA74, *patA* and *patD*	This study
pSA-patAD-cadA	pSA74, *patA*, *patD* and *cadA*	This study
pZE-gabTD	pZE12-luc, *gabT* and *gabD*	This study
pCS-gabTD	pCS27, *gab*T and *gabD*	This study
pSA-gabTD	pSA74, *gabT* and *gabD*	This study
pCS-patAD-gabTD	pCS27, *patA*, *patD*, *gabT* and *gabD*	This study
pSA-cadA-gabTD	pSA74, *cadA*, *gabT* and *gabD*	This study
pCS-patAD-gabTDP	pCS27, *patA*, *patD*, *gabT*, *gabD* and *gabP*	This study
pSA-potE	pSA74, *potE*	This study
pSA-puuP	pSA74, *puuP*	This study
pSA-potABCD	pSA74, *potABCD*	This study
pSA-potFGHI	pSA74, *potFGHI*	This study
pSA-cadA-potE	pSA74, *cadA* and *potE*	This study
pZE-lysC*-dapA*	pZE12-luc, *lysC** (T352R) and *dapA** (T118Y)	This study
pSA-cadA-ppc	pSA74, *cadA* and *ppc*	This study
pET-patA	pETDuet-1, *patA*	This study
pET-patD	pETDuet-1, *patD*	This study

*Amp^R, ampicillin resistant; Kan^R, kanamycin resistant; Gl^R, chloramphenicol resistant.

Supplementary Table 3. Oligonucleotides used in this study

**Primer**	**Sequence (5'-3')***
Overexpression	
cadA-F-*Kpn*I	GGGAAAGGTACCATGAACGTTATTGCAATATTGAATCACATG
cadA-R-*Bam*HI-*Xba*I	GGGAAATCTAGAGGATCCTTATTTTTTGCTTTCTTCTTTCAATACCTTAACGGT
patA-F-*Kpn*I	GGGAAAGGTACCATGAACAGGTTACCTTCGAGCGC
patA-R-*Sph*I	GGGAAAGCATGCTTACGCTTCTTCGACACTTACTCGC
patD-F-*Sph*I	GGGAAAGCATGCaggagatataccATGCAACATAAGTTACTGATTAACGG
patD-R-*Bam*HI-*Xba*I	GGGAAATCTAGAGGATCCTTAATGTTTAACCATGACGTGGCGGA
gabT-F-*Kpn*I	GGGAAAGGTACCATGAACAGCAATAAAGAGTTAATGCAGC
gabT-R-*Pst*I	GGGAAACTGCAGTTACTGCTTCGCCTCATCAAAACAC
gabD-F-*Pst*I	GGGAAACTGCAGaggagatataccATGAAACTTAACGACAGTAACTTATTCCG
g*abD*-R-*Bam*HI-*Xba*I	GGGAAATCTAGAGGATCCTTAAAGACCGATGCACATATATTTGATTT
gabD-R-*Eco*RI	GGGAAAGAATTCTTAAAGACCGATGCACATATATTTGATTT
gabP-F-*Eco*RI	GGGAAAGAATTCaggagatataccATGGGGCAATCATCGCAACCA
gabP-R-*Bam*HI	GGGAAAGGATCCTTAGCGCGTATTATGAACGGGTGT
potE-F-*Kpn*I	GGGAAAGGTACCATGAGTCAGGCTAAATCGAACAAAATG
potE-R-*Bam*HI-*Xba*I	GGGAAATCTAGAGGATCCTTAACCGTGTTTATTTTTCAGTTCAAAG
puuP-F-*Kpn*I	GGGAAAGGTACCATGGCTATTAATTCACCACTGAATATTGC
puuP-R-*Bam*HI-*Xba*I	GGGAAATCTAGAGGATCCTTACGTTTCACTCACCGGCGTTCTG
$P_L$lacO1-F	GAGAATTGTGAGCGGATAACAATTGAC
T1-R	CAAACAACAGATAAAACGAAAGGCCCA
patA-F-*Eco*RI	GGGAAAGAATTCGAACAGGTTACCTTCGAGCGCAT
patA-R-*Kpn*I	GGGAAAGGTACCTTACGCTTCTTCGACACTTACTCGC
patD-F-*Eco*RI	GGGAAAGAATTCGCAACATAAGTTACTGATTAACGGAGAACT
patD-R-*Kpn*I	GGGAAAGGTACCTTAATGTTTAACCATGACGTGGCGG
lysC-F-*Kpn*I-1	AAATTTGGCGGCACCAGCGTAG
lysC-F-*Kpn*I-2	GGGAAAGGTACCATGTCTGAAATTGTTGTCTCCAAATTTGGCGGCACCAGCGTAG
*lysC**-R	GGTGGTATCAAGGATTAATGCCACGCTCAC
*lysC**-F	CGTGGCATTAATCCTTGATACCACCGGTTCAA
lysC-R-*Bam*HI	GGGAAAGGATCCTTACTCAAACAAATTACTATGCAGTTTTTGC
dapA-F-*Bam*HI	GGGAAAGGATCCaggagatataccATGTTCACGGGAAGTATTGTCGCG
*dapA**-R	GATGGCTTTGAAATACTGATACAAACCTTC
*dapA**-F	GAAGGTTTGTATCAGTATTTCAAAGCCATC
dapA-R-*Xba*I	GGGAAATCTAGATTACAGCAAACCGGCATGCTTAAG
ppc-F-*Hind*III	GGGAAAAAGCTTaggagatataccATGAACGAACAATATTCCGCATTGC
ppc-R-*Pst*I	GGGAAACTGCAGTTAGCCGGTATTACGCATACCTGC

**Supplementary Table 3 continued**	
Gene knockout	
kan-F	GTGTAGGCTGGAGCTGCTTCGA
kan-R	ATGGGAATTAGCCATGGTCCATATG
kan-R-yz	AAGGTGAGATGACAGGAGATCCTG
kan-F-yz	TATCCATCATGGCTGATGCAATGC
cadA-F-up	TGATGGGCGCAAGCTCCTTCGAG
cadA-R-up	GGAACTTCGAAGCAGCTCCAGCCTACACAGTCATATCTCCAGGTAAAAAAG GCCCCT
cadA-F-down	TATTCATATGGACCATGGCTAATTCCCATTTAGCTCGTACAAGGGAAGTGGCT TGC
cadA-R-down	GGGGTAACGTAAACCAGAGAAGCATATGCG
cadA-F-yz	GCTCGCCAAGCAAACAGCTGATG
cadA-R-yz	GATGATCCTTATCACTCTGATGAACTCTGCCG
ldcC-F-up	AGGTTGTGCGTCCATTCTGTGGA
ldcC-R-up	AACTTCGAAGCAGCTCCAGCCTACACGTGTTCCTCCTGGAAAATCCTTCCTT AATCA
ldcC-F-down	TCATATGGACCATGGCTAATTCCCATCTTGCCAGAGCGGCTTCCG
ldcC-R-down	CCATTAAGCGCCAAATCCCCTTTCC
ldcC-F-yz	CGGTTATCGGTGAAGGTGGTTCTGG
ldcC-R-yz	CTGCTCATACAGTTCCAACGGCAGC
patA-F-up	ATCCAGGTCGGATAAGGCGTTTAC
patA-R-up	CTTCGAAGCAGCTCCAGCCTACACCCTGAATCCTCTCGAAAGTGTGTCA
patA-F-down	TCATATGGACCATGGCTAATTCCCATTCATGATCAGGAGTCACACCATGAC
patA-R-down	ATACCCTTTTTGATTCACCACCGTGA
patA-F-yz	AGATCATCAGCCAGTGTTTTGATGAGG
patA-R-yz	CCAGACTCACTAACATCGCTTCCATAT
gabT-F-up	AGTACGGCATCGTCGGCATCAATA
gabT-R-up	CTTCGAAGCAGCTCCAGCCTACACTGCGGCGCTGCATTAACTCTTTAT
gabT-F-down	TCATATGGACCATGGCTAATTCCCATAATCATCGCAACCACATGAGTTAGGC
gabT-R-down	CATTTCCGCCAACATCCGCATAATC
gabT-F-yz	CGCTGTTCCGCTTTAAAGATGAAGC
gabT-R-yz	ACCAGTACAACCAGCCGATGGTATA
gabP -F-up	CTACGGGGATCCAGGTCGGATAA
gabP-R-up	TCGAAGCAGCTCCAGCCTACACCCTGAATCCTCTCGAAAGTGTGTCA
gabP-F-down	CATATGGACCATGGCTAATTCCCATTCATGATCAGGAGTCACACCATGAC
gabP-R-down	TTTGATTCACCACCGTGACCAGC
gabP-F-yz	ATTGAAGACGCTCAGATCCGTCA

**Supplementary Table 3 continued**	
gabP-R-yz	CAATCGCCAGACTCACTAACATCGC
iclR-F-up	CAGCACCAGAATACGTTCATTTAACTG
iclR-*P*-R-up	TTCGAAGCAGCTCCAGCCTACACGCAATAGTTACTGAACTGATCCGATG
iclR-F-down	TCATATGGACCATGGCTAATTCCCATGAGGCAATATTCTGCCCATCATACC
iclR-R-down	ATCTCATAATGCAGCCGTAAAAGTT
iclR-F-yz	CACTTCCGGTTTACTGAGTACCAGC
iclR-R-yz	TTGGTGTTCATTTGTCTGGGCTG

*Restriction sites are underlined.
